# The effect of thymol on acetylcholine-induced contractions of the rat ileum and uterus under *ex vivo* conditions

**DOI:** 10.3389/fphar.2022.990654

**Published:** 2022-10-19

**Authors:** Blanka Premrov Bajuk, Luka Prem, Tilen Vake, Neža Žnidaršič, Tomaž Snoj

**Affiliations:** Institute of Preclinical Sciences, Veterinary Faculty, University of Ljubljana, Ljubljana, Slovenia

**Keywords:** Thymus vulgaris L, thymol, isolated organs, antispasmodic, dose-response, cholinergic system

## Abstract

Thyme (*Thymus vulgaris* L.) is a well-known medicinal plant, the aerial parts of which have long been used internally or externally as a traditional remedy for various diseases. Thyme essential oils have important pharmaceutical applications and are regularly used in the pharmaceutical, food and cosmetic industries. In folk medicine, thyme preparations are used to treat respiratory, digestive, cardiovascular and nervous disorders, as well as to relieve dysmenorrhea. Thymol, a major constituent of *Thymus vulgaris* essential oil, has been shown to affect skeletal and smooth muscle excitation and contraction. Therefore, the main objective of this study was to evaluate its effect on acetylcholine (ACh)-induced rat intestinal and uterine preparations. Isolated ileum and uterine horn preparations were placed in a 20-ml organ bath containing Tyrode or De Jalon solution and exposed to graded concentrations of ACh (0.036, 0.109, 0.36, 1.09, 3,6, 10.9, and 36 μM) and either 0.02 or 0.2 mM thymol. Additionally, the dose–response relationship of thymol impact on intestinal and uterine contraction was evaluated. Contraction changes were monitored using an isometric transducer. Thymol at the higher dose (0.2 mM) significantly reduced ACh-induced intestinal and uterine contractions. Thus, this study provides new important data on competitive actions between thymol and ACh. In the dose–response study, the IC_50_ values were calculated as 5.26 mM for the ileum and 5.35 mM for the uterus. Our results demonstrated the efficacy of thymol in reducing ileal and uterine smooth muscle contractions, thus supporting the use of thyme in traditional medicine in the treatment of digestive disorders and painful menstrual cramps.

## 1 Introduction

Thyme (*Thymus vulgaris* L) is a small aromatic medicinal plant of the Lamiaceae family that is native to Eurasia but is now widely used worldwide to flavor a variety of foods and cosmetic products (Encyclopedia Britanica: https://www.britannica.com/plant/thyme). Thyme essential oil is mainly composed of terpenes, terpene alcohols, esters and phenolic derivatives (Micucci et al., 2020). The main bioactive constituents are thymol (2-isopropyl-5-methylphenol) (46.2–67.5%) and carvacrol (5-isopropyl-2-methylphenol) (5.7–7.3.%), but their content depends on various factors, such as cultivar, geographical area, season, and cultivation practices (Hudaib and Aburjai, 2007; Boruga et al., 2014; Mancini et al., 2015; Micucci et al., 2020). Numerous studies have shown that thyme and its derivatives have anthelmintic, antimicrobial, antitussive, expectorant, antispasmodic, carminative, and diuretic effects when employed in traditional and therapeutic uses (Fachini-Queiroz et al., 2012; Salehi et al., 2018; Micucci et al., 2020). Moreover, the effect of thyme extracts and thymol itself on smooth muscle activity has been demonstrated in several *in vitro* studies. The hydroalcoholic extract of *Thymus vulgaris* suppressed the spontaneous contractions of the ileum of guinea pigs (Babaei et al., 2008). Thymus vulgaris essential oil reduced the basal contractility of guinea pig ileum and colon *ex vivo* and exerted a relaxant activity on K^+^-depolarized intestinal smooth muscle (Micucci et al., 2020). Similarly, thymol caused endothelium-independent relaxation of the rat aorta (Peixoto-Neves et al., 2010). Begrow et al. (2010) reported the antispasmodic effect of thyme extract on the tracheal and ileal contractions induced by acethylcholine (ACh), however, the authors hypothesized that thymol and carvacrol were not the only compounds with antispasmodic activity in thyme extracts. Due to its antispasmodic activity, thyme oil is also used to relieve painful cramps during menstruation (Salehi et al., 2018). A triple-blind clinical trial by Salmalian et al. (2014) conducted on 84 students showed that *Thymus vulgaris* essential oil administered as drops, was as effective as ibuprofen in reducing the severity of pain and spasm in primary dysmenorrhea. No side-effects were recorded. Interestingly, extracts of *Zataria multiflora* Boiss, which also contain thymol and carvacrol, decreased KCl- and oxytocin-induced uterine contractions (Gharib Naseri et al., 2006). On the other hand, thymol increases the absorptive capacity of the intestinal mucosa, as dietary supplementation with thymol in rabbits results in a greater villus height/crypt depth ratio in the small intestinal wall and has a beneficial effect on enterocytes (Placha et al., 2021).

The mechanism of the pharmacological activity of thymol is related to its interaction with some membrane receptors. It has been reported that low doses of thymol act as an agonist, whereas high doses antagonize α_1_-, α_2_-, and β-adrenergic receptors (Beer et al., 2007). In the review by Khazdair et al. (2018), the extract of *Zataria multiflora* Boiss, which contains thymol and carvacrol similar to thyme extracts, was reported to have adrenergic and anticholinergic effects. However, these results were not confirmed in the study by Gharib Naseri et al. (2006). Namely, these researchers demonstrated the involvement of calcium channels in the spasmolytic action of *Zataria multiflora* Boiss in rat uterus, which could support its use in traditional medicine for the relief of dysmenorrhea. The effect of thymol on skeletal muscle cells has also been reported. Tamura and Iwamoto (2004) described a controversial activity of thymol on skeletal muscle. They reported that thymol activated myosin subfragment-1 ATPase, but the isometric force of skeletal muscle fibers decreased at the same concentration. As described by Peixoto-Neves et al. (2010), thymol probably affects the transduction pathway between Ca^2+^ release from the endoplasmic reticulum and the regulation of Ca^2+^ sensitivity of the contractile system while acting at low doses.

Since the suppression of spontaneous smooth muscle contractions by thyme extracts is well documented (Babaei et al., 2008; Begrow et al., 2010) and ACh is involved in the generation of smooth muscle contractions as a neurotransmitter in the parasympathetic nervous system, the aim of this study was to investigate whether there is an antagonistic relationship between thymol, a main constituent of thyme essential oil, and ACh. The present *ex vivo* study was performed to evaluate the effects of thymol on ACh-induced contractions of isolated rat ileum and uterus.

## 2 Materials and methods

### 2.1 Animals and organ preparations

The use of animal tissues in research was approved by the Administration of the Republic of Slovenia for Food Safety, Veterinary Sector, and Plant Protection under license No. U34401-8/2018/4. Organs from 4–6-month-old female Wistar rats were used for the experiment. The rats were housed in groups of six animals under standard laboratory conditions with a controlled 12-h light-dark cycle. The rats had *ad libitum* access to food and water. A total of 14 rats were euthanized for the purpose of this study. After euthanasia with carbon dioxide and exsanguination, the abdomen of the rats was opened, and one or two 2-cm-long segments of the ileum were excised, rinsed, and placed in warmed (37 °C) Tyrode’s solution with the following composition (mM): NaCl 136.9, KCl 2.7, CaCl_2_ 1.8, MgCl_2_ 1.0, NaHCO_3_ 11.9, NaH_2_PO_4_ 0.4, and glucose 5.0 in distilled water at room temperature. Similarly, 2-cm-long segments of one or both uterine horns were collected and stored in warmed De Jalon solution (mM): NaCl 136.9, KCl 2.7, NaHCO_3_ 11.9, CaCl_2_ 1.8, and glucose 5.0 in distilled water. To evaluate the effects of thymol on organ contraction ability, seven ileal segments and ten uterine segments from six animals were used. For the dose–response experiment, eight segments of ileum and 11 segments of uterine horns from eight rats were used.

### 2.2 Substances

Acetylcholine iodide (ACh; Sigma–Aldrich, St. Louis, United States, Lot # MKCK 7580, https://www.sigmaaldrich.com/certificates/sapfs/PROD/sap/certificate_pdfs/COFA/Q14/A7000-BULKMKCK7580.pdf) with the purity ≥97% was dissolved in Tyrode or De Jalon solution (depending on the organ used) to achieve appropriate concentrations. Thymol (C_10_H_14_O) (Sigma–Aldrich, St. Louis, United States, Lot # SLBZ 9699, https://www.sigmaaldrich.com/specification-sheets/399/216/T0501-BULK.pdf) with the purity ≥98.5% was first dissolved in ethanol at a concentration of 50 mg/ml and then diluted to the selected concentration with Tyrode or De Jalon solution before starting the experiment.

### 2.3 Measurements of contractile intestine and uterine activity

A two-cm-long segment of ileum was placed vertically in a 20-ml organ bath filled with bubbled (95% O_2_/5% CO_2_) Tyrode solution at 37 °C. The rat uterine segment was also fixed as described, and the organ bath was filled with De Jalon solution. One end of the isolated organ was fixed to the bottom of the bath, and the upper end of the preparation was connected to an isometric transducer (El Unit, Belgrade, Serbia) equipped with a micrometer controller to maintain and monitor tissue tension. The preparations were equilibrated for 30 min. The nitial tension was set at 1.0 g as is reported by Aydin et al. (2009). After the equilibration period, until a stable baseline was attained, increasing concentrations of compounds were administered, and isometric muscle contraction was determined. The amplified signals were recorded using Smartplus 150 software (El Unit, Belgrade, Serbia). Responses were expressed as contraction amplitude in grams (g).

Contraction of the ileum or uterus was first monitored by administration of increasing concentrations of ACh, which stimulates smooth muscle contraction (first control). A total of six different doses of ACh were tested (0.036, 0.109, 0.36, 1.09, 3,6, 10.9, and 36 μM). After each ACh concentration, the tissue preparation was washed twice with Tyrode’s (ileum) or De Jalon (uterus) solution. To evaluate the effect of thymol, 0.02 mM of thymol was added to the solution in the organ bath 30 s before ACh-triggered contraction. The described protocol was repeated with 0.2 mM of thymol. In the final phase of the experiment, organ contractions were measured as at the beginning of the experiment only after the application of ACh (second control). The protocol for measuring uterine contractions was essentially the same, except that the Tyrode solution was replaced by De Jalon solution.

To determine the dose–response curve, increasing doses of thymol (0.166, 0.333, 1.66, 3.33, 6.66, and 9.98 mM) were administered according to a “single dose” protocol, where 3.6 μM of ACh was used to elicit ileal or uterine contraction. Each thymol dose was administered 30 s before ACh. After registration of each contraction, the organ was flushed twice with Tyrode (ileum) or De Jalon (uterus) solution.

### 2.4 Statistical analyses

Statistical analyses were performed using SPSS ver. 25 software (IBM, Chicago, United States). The distribution of the data was evaluated using Shapiro and Wilk’s test. Statistical significance between control and thymol-influenced data was evaluated using repeated-measures ANOVA with Bonferroni *post-hoc* test. Statistical significance was defined as *p* < 0.05. All results are expressed as the mean ± SE. The IC_50_ values were calculated using the on-line AAT Bioquest tool (https://www.aatbio.com/tools/ic50-calculator).

## 3 Results

### 3.1 Inhibitory effect of thymol on ACh-induced intestinal and uterine contraction

**FIGURE 1:**
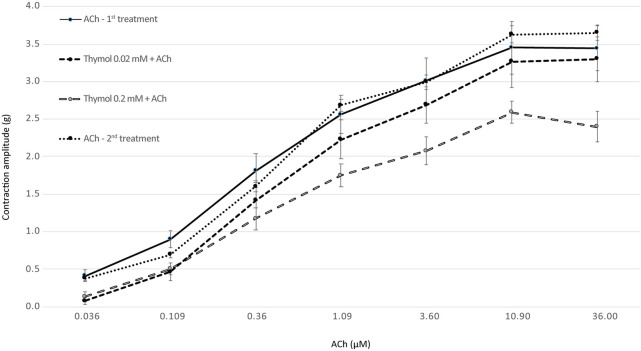
The intensity of ileal contraction elicited with increasing doses of ACh (first and second controls) and the combination of thymol (0.02 and 0.2 mM) and ACh (*N* = 7). The results are expressed as the mean ± SE of contraction amplitude (in g).

**FIGURE 2:**
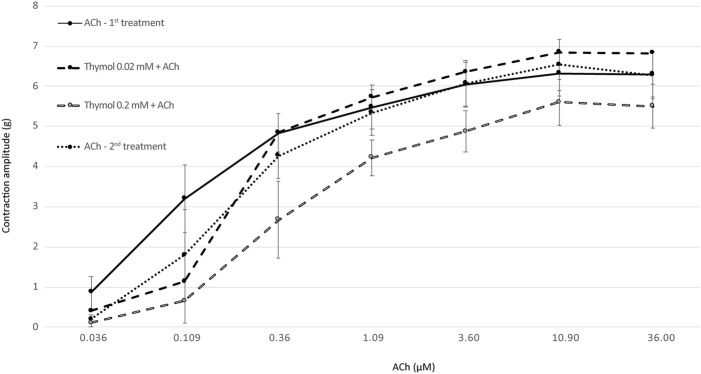
The intensity of uterine horn contraction induced with increasing doses of ACh (first and second control) and the combination of thymol (0.02 and 0.2 mM) and ACh (*N* = 10). The results are expressed as the mean ± SE of contraction amplitude (in g).

Isometric tension changes were measured in isolated rat ileum (Figure 1) and uterine horns (Figure 2).

The inhibitory effect of thymol (0.02 and 0.2 mM) on ACh-induced intestinal contraction was evaluated (Figure 1). Significantly lower (*p* < 0.05) intestinal contraction was measured after the addition of 0.02 mM thymol compared with controls only when the lowest ACh doses (0.036 and 0.109 μM) were used. Contractions induced by the combination of 0.02 mM of thymol and ACh (from 0.36 to 36 μM) did not cause statistically significant differences in the intensity of intestinal contraction. On the other hand, the administration of 0.2 mM of thymol significantly (*p* < 0.05) suppressed ileal contractions compared with controls. No significant difference in intestinal contraction was observed between the first and the second control measurements.

In a similar experiment, the effect of thymol on isolated rat uterine horns was investigated (Figure 2). No significant difference in uterine contraction was observed when a lower dose of thymol (0.02 mM) was used compared with controls. However, a significant (*p* < 0.05) decrease in uterine muscle contraction was observed when a combination of a higher dose of thymol (0.2 mM) and 0.36 or 3.6 μM ACh was administered. Although thymol at a dose of 0.2 mM decreased uterine contractions after administration of ACh at doses of 10.9 and 36 μM, the differences were not significant. Additionally, no statistically significant difference in uterine contractions was observed between the first and second controls.

### 3.2 Thymol’s dose-response effect on isolated ileum and uterus

**FIGURE 3:**
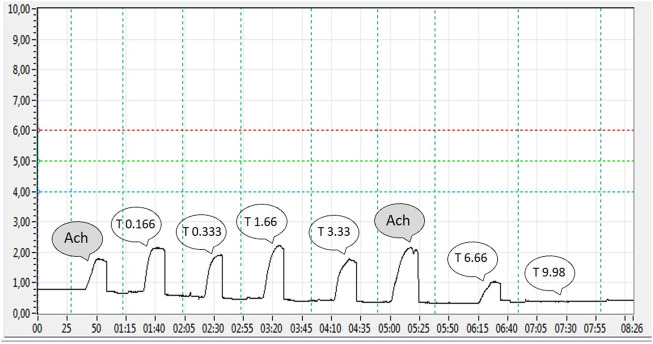
Recordings of the contraction force of a single segment of the rat ileum in the experiment to determine the dose–response relationship. Contraction of the ileum was stimulated with 3.6 μM ACh after increasing doses of thymol (T) were added (0.166, 0.333, 1.66, 3.33, 6.66, 9.98 mM).

In the second part of the study, the dose–response of thymol on the contraction of the ileum and uterus was evaluated. Different doses of thymol were added to the organ bath before the constant dose of ACh (3.6 μM). Isometric measurements in which the thymol dose was increased from 0.166 to 9.96 mM were recorded (Figure 3, SmartPlus 150 software record).

**FIGURE 4:**
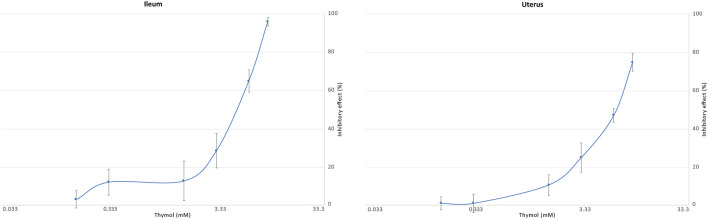
Inhibitory effect (%) of thymol (0–9.96 mM) on ACh-induced ileal (N = 8) (left) and uterine (N = 11) (right) contraction.

Increasing doses of thymol inhibited ACh-induced ileal and uterine contractions in a dose-dependent manner, which is shown in more detail in Figure 4.

The lowest inhibitory effect of thymol in some ileum and uterine preparations was observed at a dose 0.333 mM. The IC_50_ was calculated as 5.26 mM for the ileum, and 5.35 mM for the uterus. In both organs, 9.98 mM of thymol completely suppressed contraction.

## 4 Discussion

In this *ex vivo* study, the effect of thymol on ileum and uterine contractility was evaluated. Several reports describe that thyme essential oil causes relaxation or decreases contraction of various smooth muscle organs. Therefore, we investigated the inhibitory effect of thymol, the main component of thyme essential oil, on contractions of the ileum and uterus. In traditional medicine, thyme extracts are commonly used to relieve abdominal pain and dysmenorrhea. In this study, the antispasmodic effect of thymol was evaluated by measuring the ileal and uterine contractions elicited by different doses of ACh. As shown in Figures 1, 2, thymol at a higher dose (0.2 mM) significantly reduced the contractions of both organs under *ex vivo* conditions, whereas the lower dose (0.02 mM) showed an effect only when low doses of ACh were used. Thus, our study confirmed the antispasmodic effect of thymol on isolated rat ileum and uterine preparations. The results are consistent with those of other studies showing that thymol inhibits spontaneous smooth muscle contraction activity *in vitro* (Beer et al., 2007) or decreases the intensity of tracheal smooth muscle contraction induced by ACh or serotonin (Begrow et al., 2010). The exact mechanism of action of thymol was not explained in our study. Both the ileum and uterus are supplied with cholinergic and adrenergic nerves that regulate motor activity *via* muscarinic and adrenergic receptors. In gastrointestinal smooth muscle, ACh triggers contractions by activating muscarinic (M) receptors. It is generally believed that the M3 receptor plays a key role in mediating this activity in the intestine and uterus (Choppin et al., 1999; Unno et al., 2005; Kitazawa et al., 2008), although inhibitory M2 receptors are also present in these organs (Pennefather et al., 1994). Based on our experimental data, we hypothesize that thymol antagonizes muscarinic receptors. As shown in Figure 1, lower doses of thymol suppressed ACh-induced contractions only when ACh was used at the lowest doses. Ileal contractions induced by higher doses of ACh were not affected by thymol. Moreover, a high dose of thymol affected all ACh-induced contractions. These results suggest competitive binding to M receptors between thymol and ACh. Similar, although less specific effects as mentioned above, were observed when the action of thymol and ACh was tested in isolated uterine horns (Figure 2). A lower dose of thymol had no effect on ACh-induced contraction, whereas a high dose of thymol only suppressed uterine contractions triggered by low doses of ACh. Thus, compared with the ileum, the uterus responds to higher doses of thymol, probably due to the more extensive smooth muscle layer.

In the second part of our study, dose–response study of thymol was performed. Before the administration of 3.6 μM ACh, increasing doses of thymol (0.166, 0.333, 1.66, 3.33, 6.66, and 9.98 mM) were added to the organ bath. As shown in Figure 4, the percentage of contraction gradually decreased with increasing doses of thymol. Thymol suppressed ACh-induced contractions in both the ileum and uterus, suggesting that both organs might respond to thymol treatment. The IC_50_ values of thymol for the ileum and uterus were also similar (5.26 and 5.35 mM, respectively). The highest dose of thymol used (9.96 mM) irreversibly suppressed motor activity in both organs. Neither multiple flushes nor higher doses of ACh (data not shown) reactivated the organs. We cannot explain the reason for the observed strong suppression. We can only hypothesize that thymol, a liposoluble compound, could have a strong connection to the muscarinic receptor binding site. Alternatively, at this concentration, it is possible that thymol could also bind to an allosteric site in the receptor and thus alter receptor conformation. Further receptor-based studies are needed to clarify this issue.

The doses of thymol that resulted in an observable effect in this *ex vivo* study were high, much higher than the doses used for treatment (Nagoor Meeran et al., 2017). The discrepancy between the doses providing *in vitro* and *in vivo* effects has already been noted in several studies (Klm et al., 2015; Levy and Hasson, 2020).

The results of our study show that thymol, the main component of thyme (*Thymus vulgaris*) essential oil, suppresses ileal and uterine motility in an *ex vivo* experiment and thus acts as an antispasmodic agent. This finding partially confirms the antispasmodic effect of thyme preparations used in folk medicine. Given that pharmacokinetic studies have shown that thymol is rapidly absorbed after oral administration (Nagoor Meeran et al., 2017), it can exert a local effect in the digestive system and is also distributed systemically, reaching the uterus. Based on the results of our experiment and data from the literature, we believe that the beneficial effect of thyme preparations in the treatment of abdominal pain is related to thymol. However, as suggested by Begrow et al. (2010), thymol is most likely not the only compound in thyme preparations that has an antispasmodic effect.

We are aware that the results of this study did not clarify the mechanism of action of thymol. However, our study provides new important data on competitive actions between thymol and ACh that can be used in further receptor-based studies.

## 5 Conclusion

The results of this *ex vivo* study showed that thymol suppressed ACh-induced contractions of the isolated ileum and uterus of rats. The effect was dose-dependent and reversible unless a very high dose of thymol was used. A high dose of thymol irreversibly blocked the motor activity of both organs. The results suggest that thyme extracts or essential oil have an antispasmodic effect due to thymol, the main constituent of thyme preparations. Thus, our data explain why thyme and its extracts are traditionally used to treat digestive disorders and painful menstrual cramps.

## Data Availability

The raw data supporting the conclusion of this article will be made available by the authors, without undue reservation.
